# Supplementary material for: Knockout of the Amino Acid Transporter SLC6A19 and Autoimmune Diabetes Incidence in Female Non-Obese Diabetic (NOD) Mice
Source: Metabolites. 2021 Sep 29;11(10):665. doi: 10.3390/metabo11100665 (PMC8540324; doi:10.3390/metabo11100665)
Supplement: Supplementary file 1 [file metabolites-11-00665-s001.zip › metabolites-1390997-supplementary.pdf]

Supplementary Materials

CRISPR/Cas9 edited Slc6a19 deficient mouse strains Sanger sequencing chromatograms

1. Mouse samples ASD707:Maggot:::F0#6, 8, 9, 11, 12

Guide 1

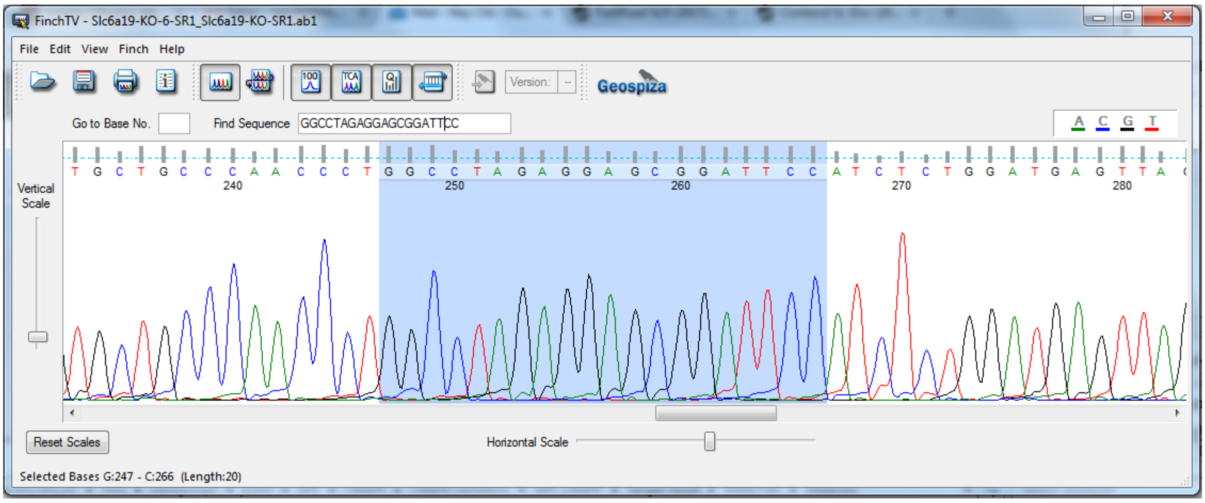

Reference AGGCTTGTGCTGCCCAAC**CCT**GGCCTAGAGGAGCGGATTCCATCTCTGGATGAGTTAGAGGTC

Allele1 AGGCTTGTGCTGCCCAAC**CCT**GGCCTAGAGGAGCGGATTCCATCTCTGGATGAGTTAGAGGTC

Allele2 AGGCTTGTGCTGCCCAAC**CCT**GGCCTAGAGGAGCGGATTCCATCTCTGGATGAGTTAGAGGTC

Guide 2

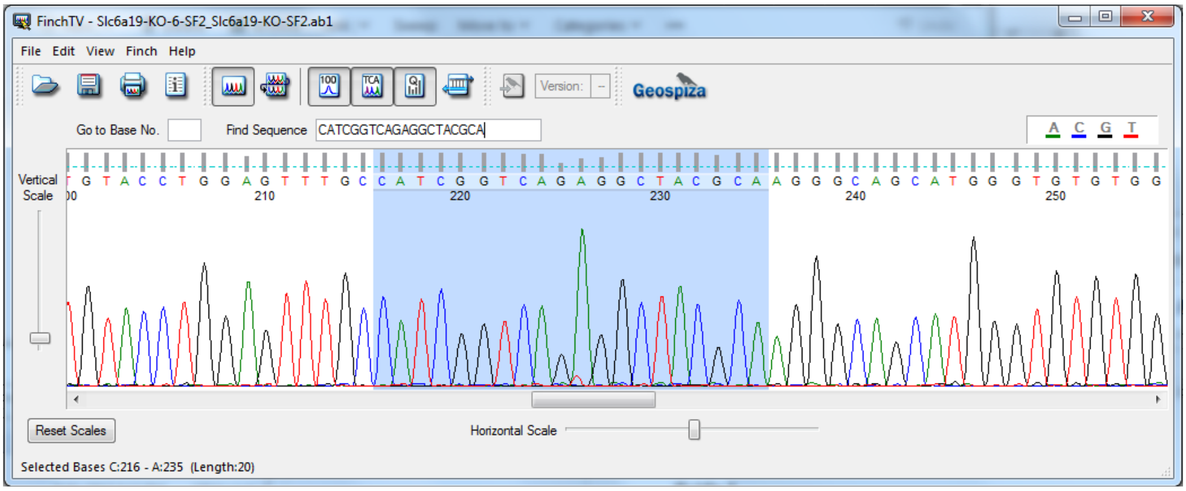

Reference TTGCTGTACCTGGAGTTTGCCATCGGTCAGAGGCTACGCA**AGG**GCAGCATGGGTGTGTGGAGC

Allele1 TTGCTGTACCTGGAGTTTGCCATCGGTCAGAGGCTACGCA**AGG**GCAGCATGGGTGTGTGGAGC

Allele2 TTGCTGTACCTGGAGTTTGCCATCGGTCAGAGGCTACGCA**AGG**GCAGCATGGGTGTGTGGAGC

Mouse sample ASD707:Maggot:::F0#6 is WT in both alleles for both guide sequences.

Mouse samples ASD707:Maggot:::F0#8, 9, 11 and 12 have the same Sanger sequencing chromatograms as mouse sample ASD707:Maggot:::F0#6.

2. Mouse samples ASD707:Maggot:::F0#7

Guide 1

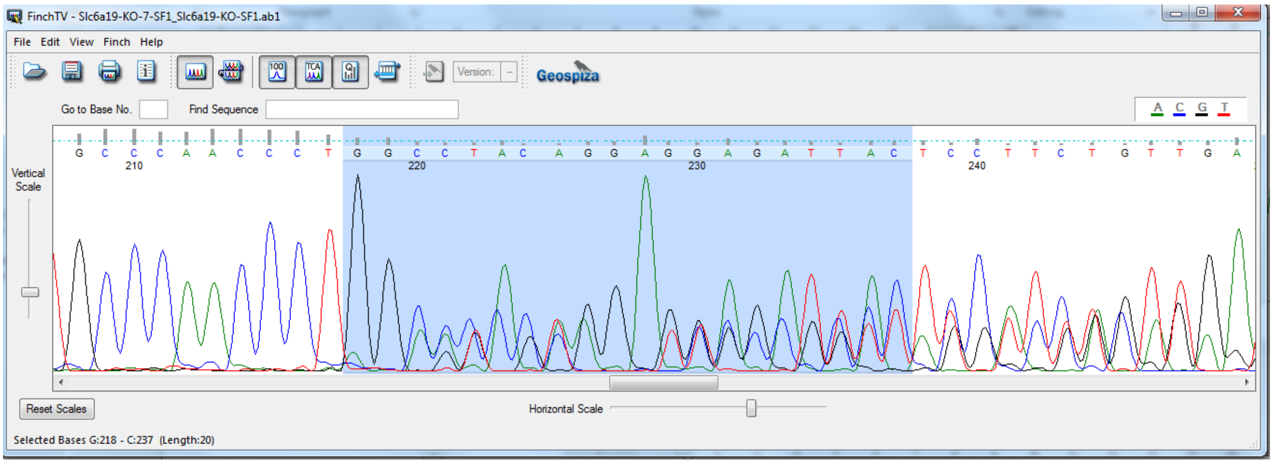

Reference GGCTTGTGCTGCCCAACC**CCT**GGCCTAGAGGAGCGGATTCCATCTCTGGATGAGTTAGAGG

Allele1 GGCTTGTGCTGCCCAACC**CCT**GG**CAC**CCTAGAGGAGCGGATTCCATCTCTGGATGAGTTAGAGG

Allele2 GGCTTGTGCTGCCCAACC**CCT**GG**-----**AGGAGCGGATTCCATCTCTGGATGAGTTAGAGG

(OR)

GGCTTGTGCTGCCCAACC**CCT**G**-----**GAGGAGCGGATTCCATCTCTGGATGAGTTAGAGG

Allele 3 GGCTTGTGCTGCCCAACC**CCT**GGCCTAGAGGAGCGGATTCCATCTCTGGATGAGTTAGAGG

Guide 2

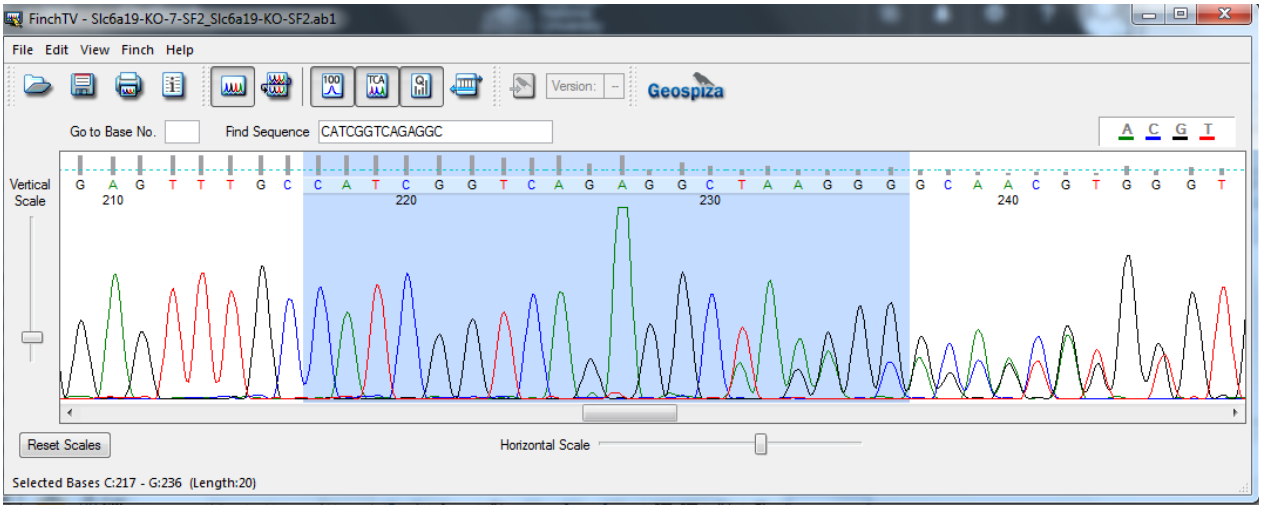

Reference TTGCTGTACCTGGAGTTTGCCATCGGTCAGAGGCTACGCA**AGG**GCAGCATGGGTGTGTGGAGC

Allele1 TTGCTGTACCTGGAGTTTGCCATCGGTCAGAGGCTA---A**AGG**GCAGCATGGGTGTGTGGAGC

Allele2 TTGCTGTACCTGGAGTTTGCCATCGGTCAGAGGC-----A**AGG**GCAGCATGGGTGTGTGGAGC

Mouse sample ASD707:Maggot:::F0#7 carries three alleles in guide 1 sequence region. The first allele carries 3bp insertion, the second allele carries 5bp deletion and the third allele is WT. In guide 2 sequence region, there are two alleles. The first allele carries 3bp deletion and the second allele carries 5bp deletion.

3. Mouse samples ASD707:Maggot:::F0#10

Guide 1

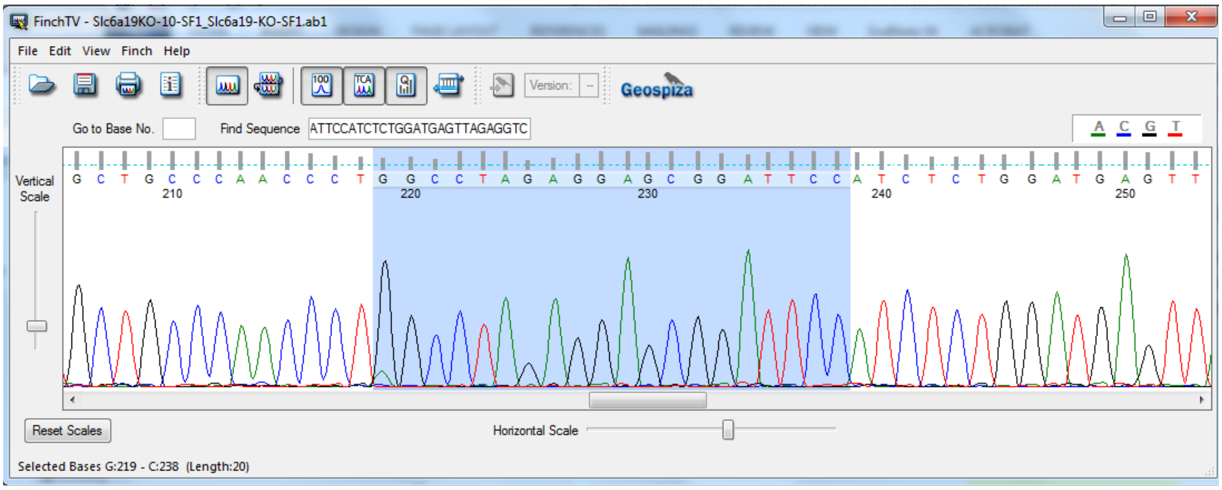

Reference AGGCTTGTGCTGCCCAAC**CCT**GGCCTAGAGGAGCGGATTCCATCTCTGGATGAGTTAGAGGTC

Allele1 AGGCTTGTGCTGCCCAAC**CCT**GGCCTAGAGGAGCGGATTCCATCTCTGGATGAGTTAGAGGTC

Allele2 AGGCTTGTGCTGCCCAAC**CCT**GGCCTAGAGGAGCGGATTCCATCTCTGGATGAGTTAGAGGTC

Guide 2

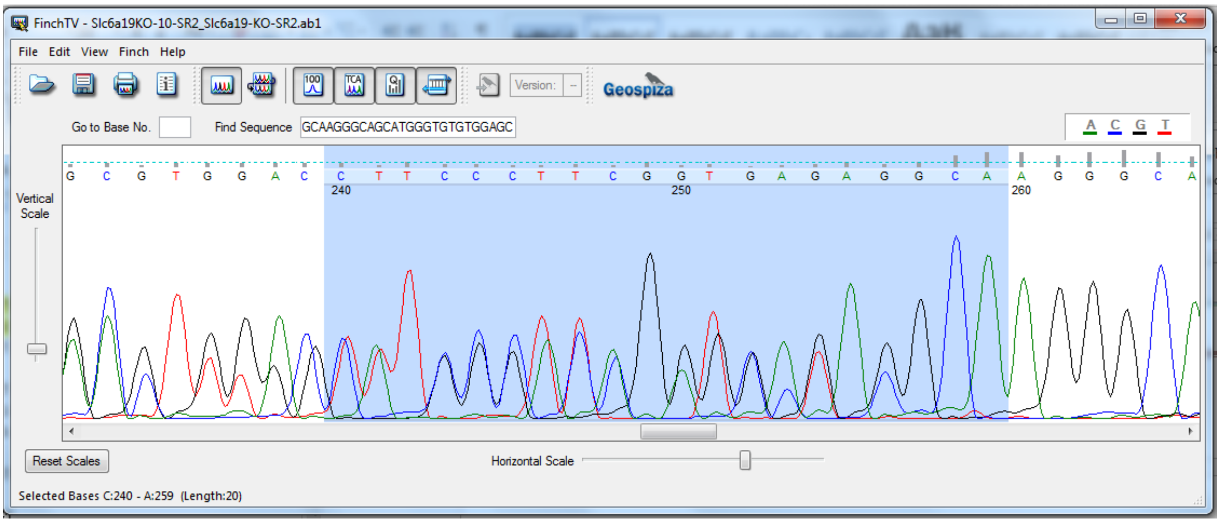

Reference TTGCTGTACCTGGAGTTTGCCATCGGTCAGAGGCTACGCA**AGG**GCAGCATGGGTGTGTGGAGC

Allele1 TTGCTGTACCTGGAGTTTGCCATCGGTCAGAG-----GCA**AGG**GCAGCATGGGTGTGTGGAGC

Allele2 TTGCTGTACCTGGAGTTTGCCATCGGTCAGAGGCTACGCA**AGG**GCAGCATGGGTGTGTGGAGC

Mouse sample ASD707:Maggot:::F0#10 is WT in both alleles in guide 1 sequence region. In guide 2 sequence region, there are two alleles. The first allele carries 5bp deletion and the second allele is WT.

4. Mouse samples ASD707:Maggot:::F0#13

Guide 1

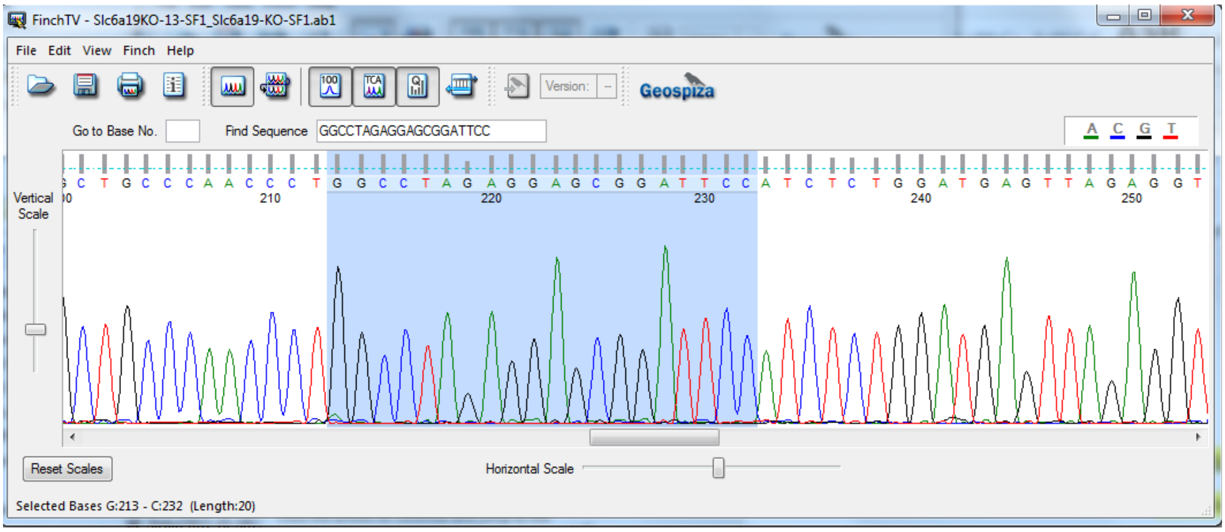

Reference AGGCTTGTGCTGCCCAAC**CCT**GGCCTAGAGGAGCGGATTCCATCTCTGGATGAGTTAGAGGTC

Allele1 AGGCTTGTGCTGCCCAAC**CCT**GGCCTAGAGGAGCGGATTCCATCTCTGGATGAGTTAGAGGTC

Allele2 AGGCTTGTGCTGCCCAAC**CCT**GGCCTAGAGGAGCGGATTCCATCTCTGGATGAGTTAGAGGTC

Guide 2

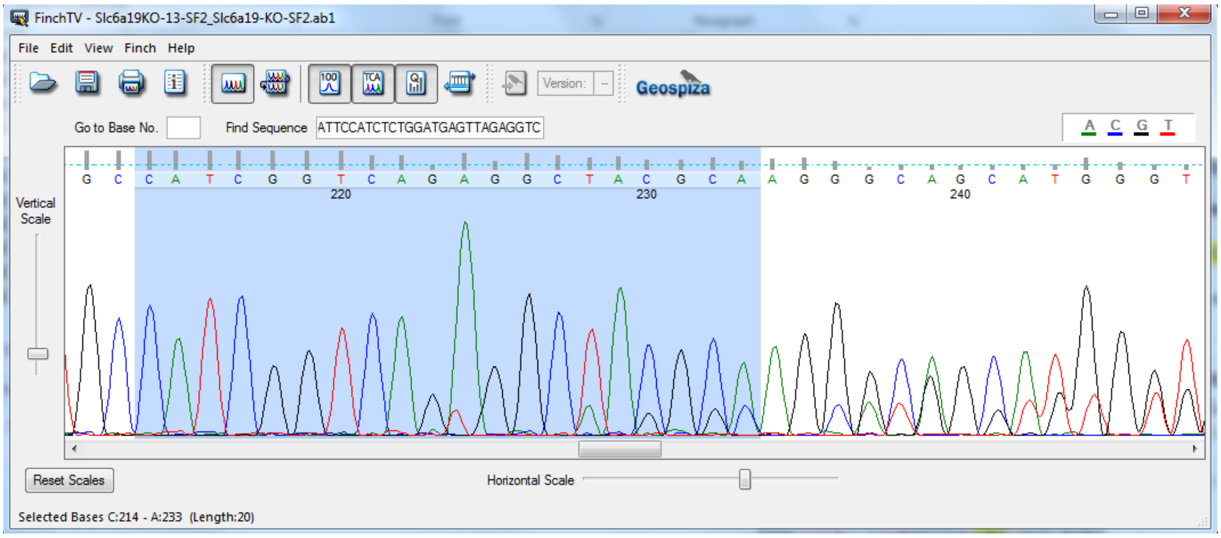

Reference TGCTGTACCTGGAGTTTGCCATCGGTCAGAGGCTACGCA**AGG**GCAGCATGGGTGTGTGGAG

Allele1 TGCTGTACCTGGAGTTTGCCATCGGTCAG-------**TG**GCA**AGG**GCAGCATGGGTGTGTGGAG

Allele2 TGCTGTACCTGGAGTTTGCCATCGGTCAGAGGCTACGCA**AGG**GCAGCATGGGTGTGTGGAG

Mouse sample ASD707:Maggot:::F0#13 is WT in both alleles in guide 1 sequence region. In guide 2 sequence region, there are two alleles. The first allele carries 7bp deletion and 2bp insertion and the second allele is WT.
